# Brain Death and Organ Donation

**DOI:** 10.31729/jnma.7367

**Published:** 2022-02-28

**Authors:** Badri Man Shrestha

**Affiliations:** 1 Sheffield Kidney Institute, Sheffield Teaching Hospitals National Health Service Trust, Sheffield, United Kingdom

Donors with brain death represent the major source of organs for transplantation and contribute to saving millions of lives of patients with organ failure. In the United Kingdom (UK) and United States of America (USA), approximately 40% of organs are recovered from brain death donors. Understanding the pathophysiology, pathways of diagnosis of brain death and ethical issues are important in the decisionmaking process for organ donation. This editorial highlights the basic science and the clinical evidence relevant to brain death and organ donation in clinical practice.

Many patients with neurological catastrophe, if not ventilated, become apnoeic, develop hypoxia and succumb from cardiac arrest. In 1959, Mollaret and Goulon, two French neurophysiologists, described a new neurological condition called "le coma dépassé" in which ventilated patients with cerebral catastrophes showed complete unresponsiveness (coma) and had loss of all brainstem reflexes, apnoea, and isoelectric readings on electroencephalography (EEG).^1^ Subsequently, in 1968, the Harvard Ad Hoc Committee on Brain Death; in 1976, the Conference of Medical Royal Colleges of the United Kingdom and several meetings and deliberations made by international experts, standardised the criteria for diagnosing brain death leading to "Uniform Determination of Death Act" which has described brain death as "irreversible cessation of all functions of the entire brain, including the brainstem".^2-4^ Currently, brain death is uniformly accepted conceptually and legally worldwide.

The term brain refers to cerebrum, brainstem and cerebellum. The primary causes of brain death are subarachnoid haemorrhage, traumatic brain injury, intracerebral haemorrhage, massive ischemic stroke, or, in rare instances, cerebral neoplasm; and the secondary cause includes global hypoxic brain injury following a cardiac arrest. Regardless of the cause, massive increases in intracranial pressure leads to compromise in cerebral circulation with secondary hypoxic brain injury; ultimately leading to cerebral circulatory arrest when the intracranial pressure exceeds the mean arterial pressure. The loss of brain function typically progresses from cerebral hemisphere to brainstem, with the medulla being last to cease functioning, as reflected in the loss of respiratory drive.^5^

Confirmation of brain death and its absolute irreversibility is paramount to maintain trust and communication with a patient's relatives to obtain approval for organ donation. For brain death testing the cause of the underlying neurological catastrophe must be clear. The process of determination of brain death includes five components: ensuring that certain prerequisites are met, neurologic examination, apnoea testing, ancillary testing (if necessary), and documentation. The confounding factors such as dysregulation of temperature, blood pressure, electrolyte levels, acid-base status and influence of drugs must be excluded.^6^

During the clinical examination, coma is established by the unresponsiveness to all noxious stimulations. Brainstem reflexes such as pupillary, corneal, oculocephalic, oculovestibular, gag, cough, and motor responses are assessed meticulously. Spinalmediated reflexes such as triple reflex must be interpreted with caution. The loss of medullary function consistent with brain death is confirmed by absence of respiratory effort in response to hypercarbia and acidosis (apnoea test). Ancillary tests are performed only when confirmation of brain death is not possible on clinical grounds in situations such as severe facial trauma or in patients with severe haemodynamic or pulmonary instability. Although EEG, evoked potentials (somatosensory, auditory and visual), transcranial Doppler ultrasonography, digital subtraction angiography, computerised tomographic angiography and magnetic resonance angiography have been utilised to assess electrical activity and cerebral perfusion; as they have false-positive and false-negative results, they should be interpreted with caution. A documentation including the name of the person performing the tests, outcomes of the tests and time of certification must be made.^7^

Brain death from any aetiology is associated with a variety of severe pathophysiological changes in cardiovascular, hormonal, and metabolic status, massive inflammatory response with a cytokine storm and complement activation, that increases immunogenicity and organ damage and adversely affects long-term survival of the transplanted organs.^8^ Organs from brain death donors are more prone to graft dysfunction and rejection when compared to organs obtained from living donors. Brain death is thus believed to be an important risk factor influencing the quality of organs before procurement and therefore, strategies should be adopted for optimisation of the donor and preservation of the organs.^5^

The UK Code of Practice for the Diagnosis and Confirmation of Death published by the Academy of the Medical Royal Colleges in 2008, is used in clinical transplant practice in the UK.^9^ Recently, a group of intensivists, neurologists, transplant physicians, and surgeons from all continents in "The World Brain Death Project", have made recommendations for maintenance of standards and consistency in determination of brain death criteria in adults and children. This document has received widespread international endorsement and serves as a guideline for professional societies involved in organ donation process.^10^

In conclusion, establishment of brain death through appropriate understanding of the pathophysiology, clinical assessment pathways and adoption of guidelines endorsed by local and international consortium is essential to encourage organ donation. Donor optimisation and organ preservation is essential for preservation of organs for better longterm transplant outcomes.
